# Progress in the application of body fluid and tissue level mRNAs-non-coding RNAs for the early diagnosis and prognostic evaluation of systemic lupus erythematosus

**DOI:** 10.3389/fimmu.2022.1020891

**Published:** 2022-10-17

**Authors:** Jiabin Liang, Fangmei Xie, Jie Feng, Chen Huang, Jian Shen, Zeping Han, Wenfeng Luo, Jinhua He, Hanwei Chen

**Affiliations:** ^1^ Central Laboratory of Guangzhou Panyu Central Hospital, Guangzhou, China; ^2^ Graduate School of Guangzhou University of Chinese Medicine, Guangzhou, China; ^3^ Radiology Department of Sun Yat-sen Memorial Hospital, Sun Yat-sen University, Guangzhou, China; ^4^ Radiology Department of Panyu Health Management Center (Panyu Rehabilitation Hospital), Guangzhou, China

**Keywords:** systemic lupus erythematosus, biomarker, mRNA, microRNA, lncRNA, snoRNA, circRNA, diagnosis

## Abstract

The diagnosis and differential classification of systemic lupus erythematosus (SLE) is difficult, especially in patients with early-onset SLE who are susceptible to systemic multi-organ damage and serious complications and have difficulties in individualized treatment. At present, diagnosis is based mainly on clinical manifestations and the detection of serological antinuclear antibodies. The pathogenesis of SLE involves multiple factors, is clinically heterogeneous, and lacks specific biomarkers. Therefore, it is necessary to identify new biomarkers for the diagnosis and subtype classification of SLE. Non-coding RNAs (ncRNAs) are composed of microRNAs, long non-coding RNAs, small nucleolar RNAs, circular RNAs, and transfer RNAs. They play an important role in the occurrence and development of diseases and are used widely in the early diagnosis and prognosis of autoimmune diseases. In this review, we focus on the research progress in the diagnosis and prognostic assessment of SLE using humoral to tissue level ncRNAs.

## Introduction

Systemic lupus erythematosus (SLE) is a chronic autoimmune disease of unknown etiology that affects nearly all organs and is characterized by immune abnormalities. The prevalence of SLE is approximately 0.02% worldwide (1) and increased nearly three-fold at the end of the 20th century (2), with Israel, the Netherlands, the United States, and other countries showing a large increase (3, 4). Organ damage occurs in 33–50% of SLE patients within 5 years of diagnosis (5). Organ damage and patient mortality also worsen over time (6, 7), seriously affecting quality of life (8, 9) and increasing medical costs (10, 11). At present, the clinically recognized diagnosis is based on symptoms and anti-double-stranded DNA (dsDNA) and anti-Smith (SM) antibody levels after excluding other diagnoses. Due to the complex manifestations of the disease, which affects many organs, there is a high misdiagnosis rate and low diagnosis rate. With the continuous improvement of biotechnology, many studies have found that epigenetic modifications play a role in the pathogenesis of SLE by altering immune homeostasis *via* the regulation of related protein-coding genes (2, 12, 13). Therefore, it is critical to identify new biomarkers that can be used for early diagnosis, increase risk stratification in screening, and reduce organ damage. Non-coding RNAs (ncRNAs) are RNA molecules that do not encode proteins but participate in gene regulation, including mainly microRNAs (miRNAs), long non-coding RNAs (lncRNAs), small nucleolar RNAs (snoRNAs), circular RNAs (circRNAs), and transfer RNAs (tRNAs). ncRNAs play an important role in the regulation of gene transcription, post-transcription, and translation. The abnormal expression of ncRNAs in plasma, saliva, urine, or tissues is associated with autoimmune and inflammatory diseases. This review summarizes the application of ncRNAs as new markers in the diagnosis and prognostic assessment of SLE from the body fluid to tissue level, in order to develop new and non-invasive biomarkers for the diagnosis of SLE.

## Current diagnosis and prognostic assessment of SLE

At present, some limitations still exist in the clinical diagnosis of SLE. For example, anti-dsDNA and anti-SM antibodies have high specificity for SLE, but anti-SM antibodies have very low sensitivity (14, 15). Anti-dsDNA and anti-SM antibodies are found in approximately 70% and 30% of SLE patients, respectively. Meanwhile, anti-Ro/SSA and anti-La/SSB antibodies are present in approximately 30% and 20% of SLE patients, respectively, but they are generally more strongly associated with Sjogren’s syndrome (14). Anti-U1 RNP antibodies are present in approximately 25% of SLE patients, but high levels of these antibodies are also found in patients with mixed connective tissue disease (14, 15). Anti-ribosomal P protein antibodies have high specificity for SLE, but low sensitivity (16).

Complete blood and classification counts, erythrocyte sedimentation rate, and C-reactive protein are commonly used in the evaluation of active SLE, but these indicators are not specific as infections can elevate their levels. Urinalysis, urine sediment examination, serum creatinine, random urine protein to creatinine ratio, and estimated glomerular filtration rate are commonly used to evaluate lupus nephritis (LN) and its severity.

Studies aiming to discover novel molecular markers based on epigenetics are being published in an endless stream. For example, DNA hypomethylation is detected in interferon (IFN)-regulated genes of lupus primitive T cells, including BST2, IFIT1, IFIT3, IFI44L, MX1, STAT1, TRIM22, and USP18 (17).Coit et al. also validated the type I IFN master regulator gene IRF7 (CD80, HERC5, IFI44, ISG15, ITGAX, and PARP12) as a specific biomarker in patients with LN (18). DNA methylated IFI44L promoter levels have been identified as a sensitive and specific biomarker for the diagnosis of SLE (19). Zhao et al. found that RFX1 inhibits Th17 cell differentiation and thus promotes SLE pathogenesis, possibly by increasing histone H3 acetylation and decreasing DNA methylation and H3K9 demethylation (20). Therefore, it is essential to find new biomarkers to replace traditional biomarkers for auxiliary diagnosis.

## Molecular pathogenesis of SLE

The etiology of SLE remains unknown. Studies suggest associations with genetic predisposition, hormonal, immune and environmental factors. The most common source of genetic susceptibility comes from major histocompatibility complex gene loci, such as class I and class II human leukocyte antigen molecules that play roles in immune surveillance, antigen processing and presentation, and complement activation during immune regulation (14). IRF5, PTPN22, SPP1, STAT4, TLR7, and TNFAIP3 are associated with the IFN-α pathway, and SLE susceptibility loci are involved in type I IFN or downstream signaling (21–24). TLR7 and TLR9 are involved in the IFN-α response (25), and immune complexes containing DNA/autoantibodies activate dendritic cells through the combined action of CD32 and TLR9 (26). Similarly, RNA-containing autoantigens activate dendritic cells *via* TLR7 (27). Both mechanisms include the stimulation of innate immunity (direct B cell production of autoantibodies) by TLR7 (which recognizes RNA) and TLR9 (which recognizes DNA), and stimulation of B cells by T cell activation in response to adaptive immunity and IFN-α (28).

The pathogenic effect of hormones on SLE may be related to their effect on the immune response. Estrogen stimulates the release of B cells, thymocytes, macrophages, CD8+ and CD4+ T cells, certain cytokines (e.g., interleukin [IL]-1), and human leukocyte antigen and endothelial cell adhesion molecule (VCAM and ICAM) expression (29, 30). Progesterone and prolactin also affect immune activity (31, 32). Progesterone reduces the proliferation of T cells and increases the levels of CD8 cells (31), and lupus episodes are associated with hyperprolactinemia (33). In addition, progesterone and high levels of estrogen can induce a Th2 response, which can lead to autoantibody production (34).

With the development of next-generation sequencing technology, an increasing number of studies have suggested that ncRNAs are associated with the occurrence and development of SLE (35). The heterogeneity of ncRNAs in the molecular pathogenesis of SLE is important for clinical practice because identifying these subtypes using different subtype-specific genetic markers can facilitate early diagnosis and personalized treatment for SLE patients.

## Candidate RNAs as SLE biomarkers

### MRNAs as SLE biomarkers

The regulatory T lymphocyte recognition marker Foxp3 plays an important role in the pathogenesis of SLE. TACI, APRIL, and BR3 mRNA levels are increased in urine samples from SLE patients (36), and urinary Foxp3 mRNA levels can be used to assess LN severity and perform risk stratification (37). Similarly, the urinary and serum levels of CCL2, CCL5, and CXCL10 are increased in active LN patients (38). There is a strong association between SIRT1 expression in urinary granules and anti-dsDNA antibodies in patients with LN (39), which are noninvasive biomarkers of SLE and LN.

TLR-3 and TLR-8 expression are associated with disease severity and susceptibility by increasing the value of C3 and C4,. IFN-α and IFN-β mRNA expression levels in whole blood may be possible biomarkers for SLE (40). MMP-2 mRNA expression is significantly increased in peripheral blood, while MMP-9 and TIMP-1 levels are significantly decreased. The mRNA expression of both correlates with SLE Disease Activity Index (SLEDAI) scores (41). The MMP/TIMP ratio curve changes with the progression of SLE (42). There is also a synergistic increase in the risk of SLE (41, 43). IL-23A and IL-18 expression are increased in SLE peripheral blood, which is positive correlation with SLEDAI scores. IL-12B, TNFA and FOXP3 expression is decreased, which are negatively correlated with SLEDAI scores and associated with LN (44).These mRNAs can be used as potential prognostic and diagnostic biomarkers.

The expression of CD13/APN mRNA is 6.12 times higher in SLE patients than in healthy subjects (45). The mRNA levels of Sema3A and NRP-1 are decreased in serum and peripheral blood mononuclear cells (PBMCs) in SLE patients, with an area under the curve (AUC) of 0.876, sensitivity of 80.6%, and specificity of 77.5% (46). The elevated serum levels of IL-36α and IL-36γ may be involved in SLE arthritis (47, 48). The expression of high-mobility group box protein 1 and TLR4 is increased in the serum and cerebrospinal fluid of patients with neuropsychiatric SLE, and may be diagnostic markers (49).

Adhesion and migration mediated by adhesion molecules are associated with the pathogenesis of SLE, and the high expression of CD44v6 mRNA in PBMCs is correlated with disease duration (50). The mRNA expression of METTL14, ALKBH5, and YTHDF2 is increased in PBMCs of SLE patients. The expression levels of IL-10, STAT4(α), and TNFSF4, are correlated with cardiovascular injury. High levels of MECP2(α) and TNF-α mRNAs are correlated with renal injury. These mRNAs may be involved in the pathogenesis of SLE (51–53).

SLC7A5 mRNA is upregulated in B and T lymphocytes in the peripheral blood of SLE patients and is associated with renal injury (54). Human endogenous retroviral E clone 4-1 mRNA is upregulated in CD4+ T cells and positively correlated with SLE disease activity (55). MZB1 mRNA expression is increased in B cells of active SLE, and MZB1 may be a therapeutic target (56). In conclusion, the abnormal expression of mRNAs in whole blood, PBMCs, serum, and urine of SLE patients has certain application prospects for the early diagnosis and prognostic assessment of the disease. A summary of these potential mRNA biomarkers is shown in **Table 1**.

**Table 1:** Potential mRNA biomarkers of SLE.

mRNA	Source	Detection method	Dysregulation	Applications/Potential mechanisms	SLE or LN patients/healthy persons	Ref.
ALKBH5	Peripheral blood	RT-PCR	Down	Correlates with C-reactive protein, N%, L%, NLR, C3, and fever	54/42	(51, 52)
CCL2, CXCL10, CD3G, CD4, TBX21, IFNG	Urine	Urine cell transcription and immunoassay	Up	Potential markers for the diagnosis of active LN		(38)
CD13/APN	Serum and peripheral blood	qRT-PCR	Up		47/44	(45)
CD44v3, CD44v6	PBMCs	RT-PCR	Up	Related to disease duration	18/18	(50)
FOXP3, IL-12B, TNFA	Peripheral blood	RT-PCR	Down	Negatively correlated with SLEDAI scores and associated with LN,Potential prognostic and diagnostic biomarkers	28/17	(44)
HMGB, TLR4	Serum and cerebrospinal fluid	RT-PCR	Up	Potential biomarkers for neuropsychiatric SLE	291/100	(49)
Human endogenous retroviral E clone 4-1	CD4+ T cells	qRT-PCR	Up	DNA hypomethylation and IL-17 release from CD4+ T cells *via* miR-302d/MBD2 in SLE and may serve as a biomarker	27/21	(55)
IFN-α, IFN-β,	Whole blood		Up	may be biomarkers for SLE	57/21	(40)
IL-18, IL-23A	Peripheral blood	RT-PCR	Up	Positive correlation with SLEDAI scores,Potential prognostic and diagnostic biomarkers	28/17	(44)
IL-36α	Peripheral blood	RT-PCR and comparative CT	Up	Associated with SLE disease activity and arthritis	49/50	(47)
IL-36α, IL-36γ	Serum	RT-PCR, ELISA	Up	May be involved in arthritis and a potential biomarker of SLE disease activity	72/63	(48)
MECP2(α), TNF -α	PBMCs	RT-PCR, ELISA	Up	Associated with kidney damage in SLE	51/50	(53)
MMP-9, TIMP-1	Whole blood and PBMCs	RT-PCR, ELISA	Down	MMP/TIMP ratio curve changes with the progression of SLE Co-existence of MMP and TIMP increases the risk of SLE MMP, TIMP, and MMP/TIMP ratio can be used as biomarkers of SLE susceptibility.	150/0 41/50 150/150	(41–43)
MTEEL14	Peripheral blood	RT-PCR	Down	Correlates with white blood cell and monocyte count	54/42	(51, 52)
MZB1	B cells	RT-PCR, flow cytometry, immunohistochemistry	Up	Potential therapeutic target for SLE oversecretion of antibodies		(56)
Sema3A	Serum and PBMCs	RT-PCR	Down	Associated with SLE disease activity and blood damage, and may be a new diagnostic biomarker for SLE	170/150	(46)
SIRT1	Urine	qRT-PCR, immunoassay of blood clots	Up	Associated with disease activity in LN and may serve as a marker of kidney damage	40/40	(39)
SLC7A5	Peripheral blood T and B lymphocytes	Flow cytometry	Up	Associated with kidney damage and may serve as a new diagnostic biomarker	50/50	(37)
SLC7A5	Peripheral blood T and B lymphocytes	Flow cytometry	Up	Associated with kidney damage and may serve as a new diagnostic biomarker		(54)
STAT4(α), TNFSF4, IL-10	PBMCs	RT-PCR, ELISA	Up	Associated with cardiovascular injury in SLE	51/50	(53)
TACI, APRIL, BR3	Urine	RT-PCR	Detectable	Potential biomarkers for LN	35/0	(36)
TIMP-2	Whole blood and PBMCs	RT-PCR, ELISA	Up	MMP/TIMP ratio curve changes with the progression of SLE Co-existence of MMP and TIMP increases the risk of SLE MMP, TIMP, and MMP/TIMP ratio can be used as biomarkers of SLE susceptibility.	150/0 41/50 150/150	(41–43)
TLR-3, TLR-8	Whole blood		Up	Associated with disease severity and susceptibility by increased the value of C3 and C4	57/21	(40)
TNFSF4	PBMCs	RT-PCR, ELISA	Up	Associated with lung and musculoskeletal injuries in SLE	51/50	(53)
YTHDF2	Peripheral blood	RT-PCR	Down	Correlates with L%, NLR, C3, and fever	54/42	(51, 52)

### MiRNAs as SLE biomarkers

miRNAs are important regulators of gene expression that have attracted much attention in recent years. They play an important negative regulatory role in the signaling process of the innate immune response and inflammatory factors. The expression of miRNA-25, miR-1273h-5p, and miR-124-3p, miR-377-3p, and miR-29b is significantly increased in PBMCs of SLE patients (57–59). The levels of circulating miR-21, miR-155, and miR-423 are significantly increased in the peripheral blood of patients with LN, while the level of miR-150 is decreased, which is involved in the pathophysiology of LN (60, 61). miR-132 expression is significantly higher in LN patients compared with non-LN SLE patients, which is helpful for the early non-invasive diagnosis of LN (62). The expression of, hsa-miR-4511, hsa-miR-1260b, hsa-miR-589-3p, and other miRNAs is increased in the peripheral blood of patients with class IV LN (63). miR-99a-3p expression is decreased in the peripheral blood of SLE patients, and miR-99a-3p binds directly to EIF4EBP1 and induces autophagy (64). The expression of miR-146a, miR-155, miR-371b-5p, and miR-5100 is increased in the peripheral blood of SLE patients (65, 66). The expression level of miR-183-5p is positively correlated with SLEDAI scores and the amount of anti-dsDNA antibodies (67).

Serum hsa-miR-766-3p regulates the PI3K-AKT-mTOR pathway in SLE patients and participates in kidney injury (68). miR-181a and miR-223 can be used as serum biomarkers to diagnose SLE and predict LN in Egyptians (69). The high expression of miR-485-5p in the serum of SLE and LN patients is positively correlated with the glomerular filtration rate and serum creatinine, proteinuria, and inflammatory cytokine levels (70). miR-203 expression is decreased in serum samples from patients with active LN, and miR-203 attenuates the activation of IL-β, IL-6, and TNF-α in TRAF6-treated HRMC and HK-2 cells (71).

miR-103, miR-150, and miR-20a levels are significantly reduced in the plasma of SLE patients, which can be used to distinguish healthy people. miR-15b, miR-19b, miR-25, and miR-93 levels are correlated with SLE disease activity, while miR-15b and miR-22 levels are correlated with the glomerular filtration rate (72). The sensitivity and specificity of miR-145 and miR-183 expression levels and anti-dsDNA antibody concentrations in the plasma of patients with different types of LN are 90.5% and 84.2%, respectively (73). The plasma levels of miR-125a, miR-146a, and miR-155 in SLE patients are significantly increased with an AUC of 0.89. miR-146a plasma levels are significantly correlated with anti-dsDNA antibody and proteinuria levels (74).

Ets-1 and miR-326 mRNA expression is increased in CD19+ B cells of SLE patients, and miR-326 may be involved in the pathogenesis of SLE by targeting Ets-1 to promote B cell differentiation (75). miR-633 levels are significantly decreased in CD4+ T cells and negatively correlated with SLEDAI scores. Deletion of miR-633 may be involved in the pathogenesis of SLE by targeting AKT1 to activate the AKT/mTOR pathway (76).

The expression of exosomal miR-21 and miR-155 is upregulated in SLE patients (77), and exosomal miR-146a is associated with the active lupus phase, proteinuria, and histological features, possibly by inhibiting the negative regulation of inflammation by IRAK1 and TRAF6 (78). The levels of miR-31, miR-107, and miR-135b-5p are significantly increased in urine exosomes from patients with proliferative lupus glomerulonephritis with an AUC of 0.783 (79), and the expression of the miRNA let-7a and miR-21 is significantly downregulated, which could be used to guide the clinical staging of patients with LN (80). The increased expression of miR-21 and miR-150 and decreased expression of miR-29c in urine exosomes and cell particles of SLE patients promote renal fibrosis by increasing pro-fibrotic molecules through the SP1 and Smad3/TGF-β pathways (81). miR-3201 and miR-1273e are downregulated in the urine of patients with LN and are involved in the pathogenesis of intravascular glomerular inflammation (82).

In conclusion, the abnormal expression of miRNAs in the blood, exosomes, and immune cells of SLE patients and LN patients has potential significance in early diagnosis and prognostic assessment. The potential miRNA biomarkers used in the diagnosis and prognostic assessment of SLE are summarized in **Table 2**.

**Table 2:** Potential miRNA biomarkers of SLE.

miRNA	Source	Detection method	Dysregulation	Applications/Potential mechanisms	SLE or LN patients/healthy persons	Ref.
hsa-miR-589-3p hsa-miR-1260b hsa-miR-4511 hsa-miR-485-5p hsa-miR-584-5p hsa-miR-543 hsa-miR-153-3p hsa-miR-6087 hsa-miR-3942-5p hsa-miR-7977 hsa-miR-323b-3p hsa-miR-4732-3p hsa-miR-6741-3p	Peripheral blood	Sequencing and database comparison	–	Potential early biomarkers of LN. May explain changes in the miRNA-mRNA regulatory network	14/7	(63)
hsa-miR-766-3p	Whole blood and serum	qRT-PCR, ELISA	Down	Potential biomarker of renal injury in SLE. Involved in SLE renal injury through the PI3K-AKT-mTOR pathway	16/0	(68)
miR-124-3p, miR-377-3p	PBMCs and serum	qRT-PCR	Up	Candidate diagnostic biomarkers for SLE	50/47	(58)
miR-132		qRT-PCR	Up	Early noninvasive diagnosis of LN	100/50	(62)
miR-146a, miR-155	Peripheral blood	qRT-PCR	Up	Biomarkers of SLE	40/32	(66)
miR-150	PBMCs	qPCR	Down	Potential biomarkers of LN	26/26	(60)
miR-183-5p	PBMCs	qPCR	Up	Potential biomarker of SLE. Positively correlates with SLEDAI scores and anti-dsDNA antibody levels	32/32	(67)
miR-21, miR-155	PBMCs	qPCR	Up	Biomarkers of SLE active nephritis	55/30	(61)
miR-21, miR-423	PBMCs	qPCR	Up	Potential biomarkers of LN	26/26	(60)
miR-25, miR-1273h-5p	PBMCs	qRT-PCR, western blotting,next-generation sequencing	Up	Potential biomarkers of SLE. miR-25 targets the expression of AMPD2 protein	3/3	(57)
miR-29b	PBMCs	circRNA sequencing	Up	Biomarker and therapeutic target for SLE		(59)
miR-371b-5p, miR-5100	Serum	qRT-PCR	Up	Biomarkers of SLE		(65)
miR-99a-3p	Peripheral blood	Second-generation high-throughput sequencing	Down	A potential therapeutic marker of SLE. Regulates and induces autophagy through the EIF4EBP1-mediated autophagy signaling pathway		(64)

### 3.3 LncRNAs as SLE biomarkers

lncRNAs are non-coding RNAs that contain more than 200 nucleotides and extensively regulate biological processes (83). The expression of lncRNAs directly affects in the occurrence and development of SLE and may be related to organ damage, clinical symptoms, and changes in biomarkers of disease activity and progression. The downregulation of the lncRNA NONHSAT101022.2 in the peripheral blood of SLE patients may increase β2-AR signal transduction *via* cis-regulation of LMBRD2, thereby inducing natural killer cells to produce high levels of IFN-γ and worsening the progression of SLE (84). The expression of AC007278.2 continues to increase in the peripheral blood of SLE patients, which regulates autoimmunity and follicular T helper cell differentiation by affecting the expression of CCR7, AZU1, and TNIP3 (85).

The expression levels of lnc-DC and GAS5 are decreased in the plasma of SLE patients, while the expression of linc0597 is increased. lnc-DC can be used to differentiate LN patients from SLE patients (86). The plasma lncRNAs GAS5, lnc7074, linc0597, linc0640, and linc5150 may be involved in the pathogenesis of SLE through the MAPK pathway (87).

The expression of the lncRNA FAS-AS1 is increased in the serum of SLE patients, which is associated with nephritis and the presence of anti-dsDNA antibodies, while the expression of the lncRNA PVT1 is decreased, which is significantly associated with oral ulcers, photosensitivity, and neurological manifestations (88). The serum levels of ANRIL and NOS3-AS A are increased in patients with atherosclerotic SLE. The expression level of ANRIL is negatively correlated with the duration of SLE, and the expression level of NOS3-AS is negatively correlated with total nitric oxide and high-density lipoprotein levels (89).

lnc0640 is overexpressed in PBMCs of SLE patients, and LNC3643 expression is significantly decreased in SLE patients with arthritis, rash, and pleurisy, and is inversely correlated with SLEDAI scores (90). H19 expression is upregulated in the serum and bone marrow mesenchymal stem cells of SLE patients and positively correlated with SLE disease activity, and thus may regulate bone marrow mesenchymal stem cell-mediated T follicular helper/regulatory T lymphocyte cell balance by inhibiting IL-2 transcription (91). The expression of lnc-FOSB-1:1 in neutrophils is significantly reduced in SLE patients and is associated with the risk of renal involvement (92). The levels of GAS5 and miR-21 are significantly increased in CD4+ T cells of SLE patients, and GAS5 is expressed at a higher level in SLE patients with ulcers (93). The lncRNA Gm20513 positively regulates the expression of H2-Aa in renal tissues of LN mice (94).

In conclusion, the abnormal expression of lncRNAs in the serum, plasma, renal tissue, PBMCs, neutrophils, and bone marrow mesenchymal stem cells of SLE patients, LN patients, and atherosclerotic SLE patients has potential significance in early diagnosis, differentiation of disease types, and assessment of prognosis. The potential lncRNA biomarkers used in the diagnosis and prognostic assessment of SLE are summarized in **Table 3**.

**Table 3:** Potential lncRNA biomarkers of SLE.

lncRNA	Source	Detection method	Dysregulation	Applications/Potential mechanisms	SLE or LN patients/healthy persons	Ref.
AC007278.2	PBMCs	qPCR, transcriptome sequencing	Up	Correlated with disease activity and severity. May enhance β2-AR signal transduction by cis-regulation of LMBRD2 and induce natural killer cells to produce high levels of IFN-γ to aggravate SLE	2/2	(85)
ANRIL, NOS3-AS, APOA1-AS	Serum	RT-PCR	Up	Predictive biomarkers of atherosclerosis in SLE	65/35	(89)
FAS-AS1	Serum	qRT-PCR	Up	Novel biomarker for SLE. Associated with nephritis and the presence of anti-dsDNA antibodies		(88)
GAS5	Serum	qRT-PCR	Down	Can be used to distinguish between LN and SLE without nephritis	77/24	(86)
GAS5	CD4+ T cells	qPCR	Up	Potential biomarker for the diagnosis and monitoring of SLE progression	45/30	(93)
GAS5, lnc7074	Serum	qRT-PCR, lncRNA microarray	Down	Potential biomarkers for SLE. Involved in the pathogenesis of SLE through the MAPK pathway, and may bind competitively with miRNAs regulating the expression of target genes	240/120	(87)
Gm20513	Kidney tissue of MRL/lpr mice	RNA-seq, qPCR	Up	Positive regulation of SLE-related h2-AA gene expression	3/3	(94)
H19	Serum, bone marrow mesenchymal stem cells	qRT-PCR	Up	Potential therapeutic target of SLE. Inhibits IL-2 transcription and regulates bone marrow mesenchymal stem cell-mediated T follicular helper/regulatory T lymphocyte balance	30/30	(91)
linc0597	Serum	qRT-PCR	Up	Can be used to distinguish between LN and SLE without nephritis	77/24	(86)
linc0597, lnc0640, linc5150	Serum	qRT-PCR, lncRNA microarray	Up	Potential biomarkers for SLE. Involved in the pathogenesis of SLE through the MAPK pathway, and may bind competitively with miRNAs regulating the expression of target genes	240/120	(87)
lnc0640	PBMCs	qRT-PCR	Up	Potential biomarkers of SLE	76/71	(90)
lnc3643	PBMCs	qRT-PCR	Down	Lower levels in SLE patients with arthritis, rash, and pleurisy than in SLE patients and negatively correlated with SLEDAI scores	76/71	(90)
lnc-DC	Serum	qRT-PCR	Down	Potential biomarkers of SLE	77/24	(86)
lnc-FOSB-1:1	Neutrophils	RNA-seq	Down	Potential biomarker for predicting recent kidney involvement. Associated with a higher risk of future kidney involvement	88/78	(92)
NONHSAT101022.2	Peripheral blood	Transcriptome sequencing	Down	Induces natural killer cells to produce high levels of IFN-γ and exacerbates the progression of SLE	77/24	(84)
PVT1	Serum	qRT-PCR	Down	Novel biomarker for SLE. Associated with oral ulceration, photosensitivity, and nervous system performance		(88)

### CircRNAs as SLE biomarkers

circRNAs are a class of highly stable ncRNAs that often show evolutionary conservation and tissue-specific expression and are helpful in diagnosing and assessing the prognosis of SLE. hsa_circ_0000479 in PBMCs of SLE patients affects the pathogenesis of SLE by reducing the expression of Wnt-16 protein (95). The expression of circPTPN22, hsa_circ_0044235, and hsa_circ_0068367 is decreased in PBMCs of SLE patients, which is negatively correlated with disease activity (96, 97). The expression levels of hsa_circRNA_100236, hsa_circRNA_101413, and hsa_circRNA_102489 are correlated with the presence of anti-dsDNA antibodies, IgG, and thrombocytopenia, respectively (97). The sensitivity and specificity of hsa_circ_0082688-hsa_circ_0008675 in PBMCs for the diagnosis of new-onset SLE with renal involvement are 79.17% and 96.64% (98), respectively. The expression of hsa_circ_0000479, hsa_circ_0082688, and hsa_circ_0082689 is increased in PBMCs of SLE patients, while hsa_circ_0000175 is decreased significantly. hsa_circ_0000479 has significant value in distinguishing SLE from rheumatoid arthritis, ankylosing spondylitis, and health composition (AUC = 0.825). hsa_circ_0000479 combined with the presence of anti-dsDNA antibodies can effectively distinguish SLE with a sensitivity of 86% and specificity of 100% (99). hsa_circ_104871 expression is significantly downregulated in the blood of SLE patients and negatively correlated with the severity of platelet involvement (100).

The downregulation of circIBTK expression in the peripheral blood of SLE patients is associated with SLEDAI scores and anti-dsDNA antibody and complement C3 levels, which may reverse miR-29b-induced DNA demethylation and AKT signaling activation (101). The downregulation of hsa_circ_0012919 expression in the peripheral blood of SLE patients can reverse the DNA hypomethylation of CD70 and CD11a in CD4+ T cells and regulate the expression of KLF13 and RANTES through miR-125a (102). circRACGAP1 expression is associated with anti-dsDNA antibody and complement C3 levels, which may affect the progression of SLE by binding to miR-22-3p to regulate the AKT signaling pathway (103). The circGARS sponge in the peripheral blood of SLE patients adsorbs miR-19 and activates the nuclear factor-κB (NF-κB) pathway by down-regulating TNFAIP3 expression (104). The expression levels of hsa_circ_0082688 and hsa_circ_0082689 are upregulated in the peripheral blood of SLE patients. The sensitivity, specificity, and accuracy of both circRNAs combined with anti-dsDNA antibody detection in the diagnosis of SLE are 95.65%, 100%, and 98.73% (99), respectively. The upregulation of hsa_circ_0002715 in whole blood of SLE patients is closely related to disease activity and the severity of hematological involvement (105). The increased expression of hsa_circ_0004156 and hsa_circ_0082626 is strongly associated with disease activity, autoantibody production, and clinical symptoms (106).

The expression of circRNA_002453 is significantly increased in the plasma of patients with LN, with a sensitivity of 0.900 and specificity of 0.841 (107). The levels of hsa_circRNA_001308 and hsa_circRNA_407176 are decreased in the plasma and PBMCs of SLE patients. The areas under the receiver operating characteristic curves for the expression of these two circRNAs were 0.662 and 0.599, respectively, in plasma, and were 0.722 and 0.806, respectively, in PBMCs (108). High T cells expressing hsa_circ_0010957 eliminate the expression of the proinflammatory protein IL-6 through the miR-125b/STAT3 signaling in SLE patients (109).

In conclusion, the abnormal expression of circRNAs in the peripheral blood, plasma, PBMCs, and T cells of SLE and LN patients is of potential significance in early diagnosis, identification of disease types, and assessment of prognosis. The potential circRNA biomarkers used in the diagnosis and prognostic assessment of SLE are summarized in **Table 4**.

**Table 4:** Potential circRNA biomarkers of SLE.

circRNA	Source	Detection method	Dysregulation	Applications/Potential mechanisms	SLE or LN patients/healthy persons	Ref.
circGARS	PBMCs	qRT-PCR, luciferase reporter gene assays, western blotting, mass spectrometry	Up	Independent biomarker of SLE progression. Regulates the expression of the YTH N6-methyladenosine RNA-binding protein 2YTHDF2 by down-regulating TNFAIP3 and the NF-κB pathway	62/62	(104)
circIBTK	PBMCs	circRNA sequencing, qRT-PCR	Down	Biomarker and therapeutic target for SLE. Reverses miR-29b-induced DNA demethylation and AKT signaling pathway activation by binding to miR-29b in SLE	42/35	(101)
circPTPN22	PBMCs	RNA-seq	Down	Diagnostic marker of SLE. Negatively correlates with disease activity and is associated with T cell activation	4/3	(96)
circRACGAP1	PBMCs	qRT-PCR, Spearman correlation analysis	Down	Therapeutic target for SLE. Regulates AKT signaling by binding to miR-22-3p		(103)
circRNA_002453	Serum	qRT-PCR, microarray	Up	Potential biomarker for the diagnosis of LN	59/27	(107)
hsa_circ_0000479	PBMCs	Next-generation sequencing, RT-PCR	Up	Novel biomarker for the diagnosis of SLE. Reduces the expression of WNT-16 protein to affect the incidence of SLE	64/58	(95)
hsa_circ_0000479	PBMCs	circRNA microarray, qRT-PCR	Up	In conjunction with anti-dsDNA antibody test for the diagnosis of SLE	50/45	(99)
hsa_circ_0002715	Whole blood	circRNA microarray, qRT-PCR	Up	Potential diagnostic and differential marker for SLE. Related to the severity of disease activity and blood system involvement	76/33	(105)
hsa_circ_0004156, hsa_circ_0082626	Whole blood	qRT-PCR	Up	Potential diagnostic and differential diagnostic markers of SLE and indicators to assess disease severity and activity	47/32	(106)
hsa_circ_0010957	CD4+ T cells	RT-qPCR, ELISA, reporter gene assays, western blotting	Up	Potential biomarker and therapeutic target for SLE. Mediates miR-125B/STAT3 signaling and eliminates the pro-inflammatory effect of IL-6	30/25	(109)
hsa_circ_0012919	CD4+ T cells	circRNA chip, luciferase reporter gene assays, FISH	Down	Biomarker of SLE. Increases DNMT1 expression and decreases CD70 and CD11a expression, and reverses the DNA hypomethylation of CD11a and CD70 in CD4+ T cells of SLE patients	28/18	(102)
hsa_circ_0044235, hsa_circ_0068367	PBMCs	qRT-PCR, microarray	Down	Biomarkers for the diagnosis of SLE	10/10	(97)
hsa_circ_0082688	Peripheral blood	circRNA microarray, qRT-PCR	Up	Potential biomarker for SLE diagnosis. May also serve as a potential biomarker for the diagnosis of SLE with renal involvement	6/3	(98)
hsa_circ_0082688 hsa_circ_0008675	PBMCs	circRNA microarray, qRT-PCR	Up	Potential biomarkers for the diagnosis of SLE with renal involvement	50/45	(99)
hsa_circ_0082689	Peripheral blood	circRNA microarray, qRT-PCR	Up	Potential biomarker for SLE diagnosis	6/3	(98)
hsa_circ_104871	PBMCs	qRT-PCR	Down	Potential diagnostic and differential diagnostic marker of SLE. Negatively correlates with the severity of SLE hematological platelet involvement	70/70	(100)
hsa_circRNA_100236, hsa_circRNA_102489, hsa_circRNA_101413	PBMCs	qRT-PCR	Up	Novel biomarkers for the diagnosis of SLE. Associated with the presence of anti-dsDNA antibodies, thrombocytopenia, and IgG	10/10	(97)
hsa_circRNA_407176, hsa_circRNA_001308	Serum and PBMCs	qRT-PCR	Down	Potential biomarkers of SLE	122/102 26/26	(108)

### TRNAs, tRNA-derived fragments, and tRNA stress-induced small RNAs as SLE biomarkers

The main role of tRNAs is to deliver amino acids to ribosomes for protein synthesis under the direction of mRNAs. A recent study has shown that dysregulated tRNAs and tRNA-derived small RNAs (tsRNAs) are involved in the pathophysiology of human diseases (110). A total of 160 tsRNAs were identified by high-throughput sequencing in LN renal tissue, of which 79 were upregulated and 81 were downregulated. Bioinformatics analysis predicts that tsRNAs may play a role in the pathogenesis of LN through cell adhesion molecules as well as the B-cell receptor, PI3K-AKT, and MAPK signaling pathways (111). A total of 101 tRNAs and 355 tsRNAs were differentially expressed in PBMCs of SLE patients. Gene Ontology analysis and Kyoto Encyclopedia of Genes and Genomes pathway analysis revaled that the changes in the target genes of tRNAs were most enriched in SLE. The target genes altered by tsRNAs were most enriched in Th1 and Th2 cell differentiation, the T cell receptor signaling pathway, and primary immune deficiency. These pathways may play a role in the development of SLE (112). Meanwhile, 482 differentially expressed tRFs were identified in the CD4+ T cells of SLE patients, among which tRF-3009 expression was upregulated and positively correlated with serum IFN-α levels, active LN, and SLEDAI scores (113). The expression of tRF-His-GTG-1 was significantly upregulated in the serum of SLE patients. The sensitivity and specificity of tRF-His-GTG-1 combined with the presence of anti-dsDNA antibodies for the diagnosis of SLE were 83.72% and 94.19%, respectively (114).

In conclusion, the abnormal expression of tRNAs, tsRNAs, tRFs, and tiRNAs in peripheral blood and kidney tissue of SLE patients and LN patients has potential significance in the early diagnosis and prognostic assessment of the disease. The potential tRNA biomarkers used in the diagnosis and prognostic assessment of SLE are summarized in **Table 5**.

**Table 5:** Potential tRNA and snoRNA biomarkers of SLE.

tRNA	Source	Detection method	Dysregulation	Applications/Potential mechanisms	SLE or LN patients/healthy persons	Ref.
SNORA12	T cells		Up	Negatively correlates with SLEDAI scores, changes the expression of CD69 and IFN-γ secretion, and affects the development of SLE.	192/109	(115)
tRF-3009	CD4+ T cells	High-throughput sequencing	Up	Positive correlations with SLEDAI scores, active LN, and serum IFN-α levels, and is associated with the IFN-α and oxidative phosphorylation (OXPHOS) pathways	23/17	(113)
tRF-His-GTG-1	Serum	RT-PCR	Up	Biomarker for the diagnosis and prediction of LN		(116)

### SnoRNAs as SLE biomarkers

snoRNAs have been shown to direct the chemical modifications of rRNAs (116). In addition, snoRNA-derived fragments have been shown to regulate gene expression and play a role in disease progression (117, 118). Decreased H/ACA box nucleolar RNA12 (SNORA12) expression in the T cells of SLE patients is negatively correlated with the disease activity score. SNORA12 overexpression decreases the expression of histone cluster 1H4 family member K and changes the expression of CD69. IFN-γ secretion is then inhibited and affects the development of SLE (115). The potential snoRNA biomarker used in the diagnosis and prognostic assessment of SLE are summarized in **Table 5**.

## Conclusion and prospects

SLE is a heterogeneous autoimmune disease with a mortality rate that is still 2–5 times that of the general population (119, 120). According to the Centers for Disease Control and Prevention mortality data from 2000 to 2015, SLE is among the top 20 leading causes of death in women aged 5–64 years (121). It can affect almost all organs, and patients often undergo multiple remissions and relapses with varying severity, making it sometimes difficult to make a definitive diagnosis. Delayed diagnosis is one of the most critical issues in SLE. Although referral rheumatologists have generally improved in differentiating SLE from multiple immune diseases, the activity of SLE, the diagnosis of LN and disease progression, and even the prediction of target organ damage are not well established. Increased disease activity is sufficient to alter treatment regimens (122–124). However, screening is now routinely performed in many countries, and techniques are under development that will be less invasive and will complement or perhaps even replace the current comprehensive diagnostic approach to SLE. In these new techniques, additional progress will be required to realize early diagnosis and to monitor the disease activity of SLE and LN as well as to identify potential therapeutic targets. Increasing evidence indicates that ncRNAs play a crucial role in the development and progression of SLE. The advent of high-throughput sequencing technology has also further advanced our understanding of the epigenetics and transcriptomics of SLE and other immune diseases. In this review, we described the potential of various RNAs to serve as biomarkers for SLE, perhaps allowing liquid biopsies to replace tissue samples and cell line models. In addition, the simultaneous application of multiple RNA biomarkers has the potential to enhance the sensitivity and specificity of diagnosis and prognosis. The ability to of these biomarkers to detect RNAs in various body fluids is advantageous because it allows for non-invasive diagnosis. There are many studies on RNA biomarkers for SLE, but no unified opinion. Ongoing research aims to determine which noninvasive diagnostic biomarkers for SLE are feasible and cost-effective, to understand which biomarkers can better assess patient outcomes, and to identify more personalized therapeutic targets.

## Author contributions

JL, FX, and JF contributed equally to this work. JL and JF: organized the literature and original draft writing; ZH and JS: contributed to literature retrieval and data collation; WL, CH and JS: contributed to manuscript revision; HC and JH: conception, writing review, and approval of the submitted version. All authors contributed to the article and approved the submitted version.

## Funding

This work was supported by the Medical Science and Technology Research Project of Guangdong Province (Nos. A2022524; A2020304), Science and Technology Program of Guangzhou (Nos. 202102080532; 202002030032; 202002020023), Health Commission Program of Guangzhou (20212A010025; 20201A010085), Science and Technology Project of Panyu, Guangzhou (2021-Z04-053; 2020-Z04-026; 2019-Z04-02), and Scientific Research Project of Guangzhou Panyu Central Hospital (Nos. 2022Y002; 2021Y004; 2021Y002).

## Acknowledgments

We gratefully thank all the editors and reviewers for their valuable contributions.

## Conflict of interest

The authors declare that the research was conducted in the absence of any commercial or financial relationships that could be construed as a potential conflict of interest.

## Publisher’s note

All claims expressed in this article are solely those of the authors and do not necessarily represent those of their affiliated organizations, or those of the publisher, the editors and the reviewers. Any product that may be evaluated in this article, or claim that may be made by its manufacturer, is not guaranteed or endorsed by the publisher.

## Glossary

**Table:**

AKT1	Serine/threonine-protein kinase akt-1
ALKBH5	RNA demethylase ALKBH5
ANRIL	Antisense non-coding RNA in the INK4 locus
APRIL	A proliferation-inducing ligand
AUC	Area under the curve
AZU1	Azurocidin 1
BR3	Balbiani ring protein 3
BST2	Bone marrow stromal antigen 2
CCL2	C-C motif chemokine 2
CCL5	C-C motif chemokine 5
CCR7	C-C motif chemokine receptor 7
circRNA	Circular RNA
CXCL10	C-X-C motif chemokine 10
dsDNA	Double-stranded DNA
Ets-1	ETS proto-oncogene 1
Foxp3	Forkhead box P3
GAS5	Growth arrest-specific 5
H2-Aa	H-2 class II histocompatibility antigen, A alpha chain
H3K9	Ninth lysine on histone H3
HERC5	HECT And RLD domain containing E3 ubiquitin protein ligase 5
HK-2	Human kidney-2
HRMC	Human renal mesangial cells
ICAM	Intercellular cell adhesion molecule
IFI44	Interferon-induced protein 44
IFI44L	Interferon-induced protein 44-like
IFIT1	Interferon-induced protein with tetratricopeptide repeats 1
IFIT3	Interferon-induced protein with tetratricopeptide repeats 3
IFN	Interferon
IFN-γ	Interferon gamma
IL-1	Interleukin-1
IL-10	Interleukin-10
IL-12B	Interleukin-12 subunit beta
IL-18	Interleukin-18
IL-36α	Interleukin-36 alpha
IL-36γ	Interleukin-36 gamma
IL-6	Interleukin-6
IL-β	Interleukin 1 beta
IRAK1	Interleukin-1 receptor associated kinase 1
IRF5	Interferon regulatory factor 5
IRF7	Interferon regulatory factor 7
ISG15	Interferon-stimulated gene 15
ITGAX	Integrin alpha-X
KLF13	Krueppel-like factor 13
LMBRD2	G-protein coupled receptor-associated protein LMBRD2
LN	Lupus nephritis
lncRNA	Long non-coding RNA
MAPK	Mitogen- activated protein kinase 1
MECP2	Methyl-CpG-binding protein 2
METTL14	Methyltransferase 14
miRNA	MicroRNA
MMP-2	Matrix metallopeptidase 2
MMP-9	Matrix metallopeptidase 9
mTOR	Mammalian target of rapamycin
MX1	Interferon-induced GTP-binding protein Mx1
MZB1	Marginal zone B- and B1-cell-specific protein
ncRNA	Non-coding RNA
NF-κB	Nuclear factor-κB
NOS3-AS	Nitric oxide synthase 3-AS
NRP-1	Neuropilin-1
PARP12	Poly(ADP-ribose) polymerase 12
PBMC	Peripheral blood mononuclear cell
PI3K	Phosphoinositide-3-kinase
PTPN22	Protein tyrosine phosphatase non-receptor type 22
RANTES	Regulated upon activation normal T cell expressed secreted factor
RFX1	Regulatory factor X1
Sema3A	Semaphorin 3A
SIRT1	Sirtuin-1
SLC7A5	Solute carrier family 7 member 5
SLE	Systemic lupus erythematosus
SLEDAI	Disease activity index
SM	Smith
snoRNA	Small nucleolar RNA
SP1	Transcription factor Sp1
SPP1	Secreted phosphoprotein 1
STAT1	Signal transducer and activator of transcription 1
STAT4	Signal transducer and activator of transcription 4
TACI	Transmembrane activator and CAML interactor
Th17	T helper cell 17
Th2	T helper cell 2
TIMP-1	Metalloproteinase inhibitor 1
tiRNAs	tRNA stress-induced small RNAs
TLR7	Toll-like receptor 7
TLR9	Toll-like receptor 7
TNFA	Tumor necrosis factor alpha
TNFAIP3	TNF alpha induced protein 3
TNFSF4	Tumor necrosis factor ligand superfamily member 4
TNIP3	TNFAIP3 interacting protein 3
TRAF6	TNF receptor-associated factor 6
tRFs	tRNA-derived fragments
TRIM22	Tripartite motif-containing 22
tRNA	Transfer RNA
tsRNAs	tRNA-derived small RNAs
U1 RNP	U1 ribonucleoprotein
USP18	Ubiquitin-specific peptidase 18
VCAM	Vascular cell adhesion molecule
YTHDF2	YTH domain-containing family protein 2
